# Safety Profile of Oxaliplatin in 3,687 Patients With Cancer in China: A Post-Marketing Surveillance Study

**DOI:** 10.3389/fonc.2021.757196

**Published:** 2021-10-21

**Authors:** Zaoqin Yu, Rui Huang, Li Zhao, Ximin Wang, Xiaofang Shangguan, Wei Li, Min Li, Xianguo Yin, Chengliang Zhang, Dong Liu

**Affiliations:** ^1^ Department of Pharmacy, Tongji Hospital, Tongji Medical College, Huazhong University of Science and Technology, Wuhan, China; ^2^ School of Pharmacy, Tongji Medical College, Huazhong University of Science and Technology, Wuhan, China; ^3^ Hubei Center for Adverse Drug Reaction Monitoring, Wuhan, China

**Keywords:** oxaliplatin, cancer, post-marketing surveillance, safety, Chinese

## Abstract

**Background:**

Oxaliplatin (OXA), a third-generation platinum derivative, has become one of the main chemotherapeutic drugs for colorectal cancer and other cancers, but reports of adverse reactions are also increasing with the extensive application of OXA. In this study, post-marketing surveillance was carried out to investigate the safety profile of OXA in a real-world setting in Chinese cancer patients to provide a reference for the rational application of OXA.

**Methods:**

All patients with cancer who received OXA-based chemotherapy in 10 tertiary hospitals in Hubei Province, China, between May 2016 and November 2016 were enrolled. A central registration method was used to document patients’ demographics, clinical use, and any incidence of adverse reactions to OXA. All adverse drug reactions (ADRs) were collected and analyzed to assess causality, severity, treatment, and outcome.

**Results:**

In total, 3687 patients were enrolled in this study. Approximately 64.6% of the patients were male, and 68.8% were aged 50-70 years, with a mean age of 55.3 years. The proportions of patients diagnosed with colorectal and gastric cancers were 59.3% and 31.6%, respectively. In this study, the overall incidence of ADRs and serious ADRs was 42.7% and 1.3%, respectively. The most common ADRs were gastrointestinal disorders (25.7%), blood disorders (21.1%), and peripheral nervous system disorders (8.0%). The serious ADRs identified were hypersensitivity reactions, thrombocytopenia, abnormal hepatic function, and leukopenia/neutropenia. The median onset of gastrointestinal toxicity, myelosuppression, peripheral neurotoxicity, and abnormal hepatic function was 1 d, 5 d, 1 d, and 14 d, respectively. The majority (84.7%) of hypersensitivity reactions were mild to moderate, and the median time to onset of these reactions was within the first 20 min of OXA infusion. Almost 88.0% of patients who experienced ADRs recovered or improved with treatment.

**Conclusion:**

Our data suggest that OXA-induced ADRs are very common in Chinese patients with cancer; however, more attention should be paid to hypersensitivity reactions caused by OXA. This study provides a valuable reference regarding the safe application of OXA in a real-world setting.

## Introduction

Oxaliplatin (OXA) is a third-generation platinum-based anti-tumor drug that inhibits DNA and protein synthesis by forming intra- and inter-strand DNA platinum adducts, leading to tumor growth inhibition and apoptosis (1). OXA has better efficacy and lower toxicity than cisplatin and carboplatin (2) and is extensively used in various tumors, including colorectal, gastroesophageal, pancreatic, biliary, gynecologic malignancies, lung cancer and head and neck cancers (3–5). Studies have demonstrated that OXA combined with 5-fluorouracil (5-FU) and leucovorin increases survival and reduces the risk of recurrence in patients with colorectal cancer (CRC) (6, 7). Nowadays, OXA combined with 5-FU and leucovorin (FOLFOX) or with capecitabine (XELOX) has emerged as the standard regimen of adjuvant chemotherapy for stage III CRC and stage II CRC with high-risk factors and as the first-line regimen for metastatic CRC (8, 9).

With the extensive clinical application of OXA, adverse reactions towards OXA have also been reported in recent years, mainly including gastrointestinal side effects, hematologic toxicities, and dose-limiting peripheral neurotoxicity (10–12). Moreover, the reports on OXA-related hypersensitivity reactions (HSRs) are increasing gradually (13). Although OXA has been on the market for over 20 years, and there have been a few reports on its side effects, comprehensive safety profiles of OXA in large-scale populations in the real world have rarely been produced. The MOSAIC trial, a large randomized multi-institution randomized trial, only included over 1,100 patients who were receiving adjuvant FOLFOX chemotherapy for colorectal cancer (14). Thus, it is necessary to evaluate the post-marketing safety of OXA further to strengthen pharmacovigilance. In this article, a multicenter prospective study was carried out in 10 tertiary hospitals in Hubei province, China, to evaluate the post-marketing safety profile of OXA. We aimed to investigate the clinical use of OXA, the incidence of adverse reactions, time to onset, clinical manifestations, treatments, and outcomes to provide a reference for medical decision-making and the rational application of OXA.

## Methods

### Study Design and Patients

This multicenter observational study was conducted in 10 tertiary hospitals in Hubei Province, China, between May 2016 and November 2016. All patients with malignant tumors treated with OXA were enrolled in this study. All patients were prospectively registered upon initiation of OXA treatment by documenting the patients’ demographics, clinical application, and adverse reactions of OXA using a central monitor method.

The investigators received unified and standardized project training according to research work manual and case report forms (CRF) (**Supplementary Table 1**) before the study to ensure complete registration and quality data. Meanwhile, each subcenter designated special personnel (including one oncologist and one clinical pharmacist) responsible for the collection and filing of CRF. This study was conducted in accordance with the Drug Reevaluation Regulations and Guiding Principles from the Drug Evaluation Center of the State Food and Drug Administration and was approved by the Ethics Committee of Tongji Medical College, Huazhong University of Science and Technology (No.TJIRB20160504). All participants were briefed and have provided written informed consent before completing the survey.

### Safety Assessment

OXA-induced ADRs were collected through patients’ self-reports (especially some symptoms such as rash, cough, nausea, vomiting, abdominal pain, numbness and dizziness) during ward round and patient education as well as lab results (such as vital signs, blood routine, liver and kidney function) from HIS system, and the frequency was usually 3 times a week. The investigators needed to report all adverse reactions that occurred after OXA treatment, concomitant medications used, time to onset, symptoms, administered treatments, and outcomes. All reported ADRs were recorded according to the Provisions of Adverse Drug Reaction Reporting and Monitoring with the clinical pharmacists’ assessment of causality. This adverse reaction can be submitted only when the ADR correlation evaluation with OXA is possible or above. The severity of OXA-induced ADRs was classified according to the National Cancer Institute Common Criteria (NCI-CTCAE v5.0), and grade 3 and above adverse reactions were defined as serious ADRs. ADRs were classified using the preferred terms and system organ classes from the World Health Organization (WHO) Glossary of Adverse Drug Reactions. Additional information, such as the results of laboratory tests, was also recorded for serious ADRs.

### Statistical Analysis

More than 3000 patients receiving OXA were enrolled to detect an ADR in one out of every 1000 patients with a probability of ≥95%. The collected data were verified and encoded by special personnel. All data analyses were performed using SPSS version 24.0 (SPSS Inc. Chicago, IL, USA). Descriptive analysis was used to evaluate patients’ demographics (such as sex and age), clinical characteristics such as Karnofsky performance status (KPS), diagnosis, clinical use of OXA, and the incidence of adverse reactions, time to onset, symptoms, administered treatments, and outcomes.

## Results

### Patient Demographics

A total of 3775 case report forms were enrolled between May 1, 2016, and November 31, 2016. Eighty-eight reports were excluded due to duplication; thus, 3687 patients were enrolled for this safety study. **Table 1** describes the patient demographics and clinical characteristics. Nearly 64.6% of patients were male, and most patients were between 40 and 70 years old (86.5%), with a mean (SD) age of 55.3 ( ± 10.6) years. Most patients had KPS scores of 80 (40.4%) and 90 (36.7%). Almost all (96.2%) patients were diagnosed with gastrointestinal cancer, with 59.3% and 31.6% of these patients diagnosed with colorectal and gastric cancer, respectively. Nearly two-thirds (61.7%) of the patients used the FOLFOX regimen, followed by the XELOX (19.5%) and SOX (S-1 plus OXA) (7.5%) regimens. About 25.5% of the patients had other diseases such as hypertension, hyperlipidemia, diabetes, coronary heart disease, kidney stones, and tuberculosis. Only 0.8% of the patients had a history of allergies, and 5.9% had a previous history of ADRs, mainly caused by antibiotics.

**Table 1:** Patient demographics and baseline characteristics.

Characteristics	n = 3687 n (%)
Sex	
Male	2382 (64.6)
Female	1305 (35.4)
Age (years)	
20-29	112 (3.0)
30-39	185 (5.0)
40-49	651 (17.7)
50-59	1326 (36.0)
60-69	1211 (32.8)
≥70	202 (5.5)
KPS score	
100	379 (10.3)
95	17 (0.5)
90	1352 (36.7)
85	324 (8.8)
80	1490 (40.4)
70	94 (2.5)
60	11 (0.3)
Missing and wrong entry	4 (0.1)
Histology or cytology	
Colorectal cancer	2187 (59.3)
Gastric cancer	1166 (31.6)
Liver cancer	114 (3.1)
Esophageal cancer	56 (1.5)
Pancreatic cancer	24 (0.7)
other	140 (3.8)
Complication	
Yes	940 (25.5)
No	2747 (74.5)
OXA- based chemotherapy	
FOLFOX	2274 (61.7)
FOLFOXIRI	10 (0.3)
XELOX	718 (19.5)
GEMOX	39 (1.1)
SOX	278 (7.5)
EOX	2 (0.1)
Others	366 (9.3)
History of Allergy	
Yes	31 (0.8)
No	3656 (99.2)
Previous history of ADRs	
Yes	219 (5.9)
No	2915 (79.1)
Unknown	553 (15.0)

### Incidence of ADRs and Serious ADRs

**Figure 1:**
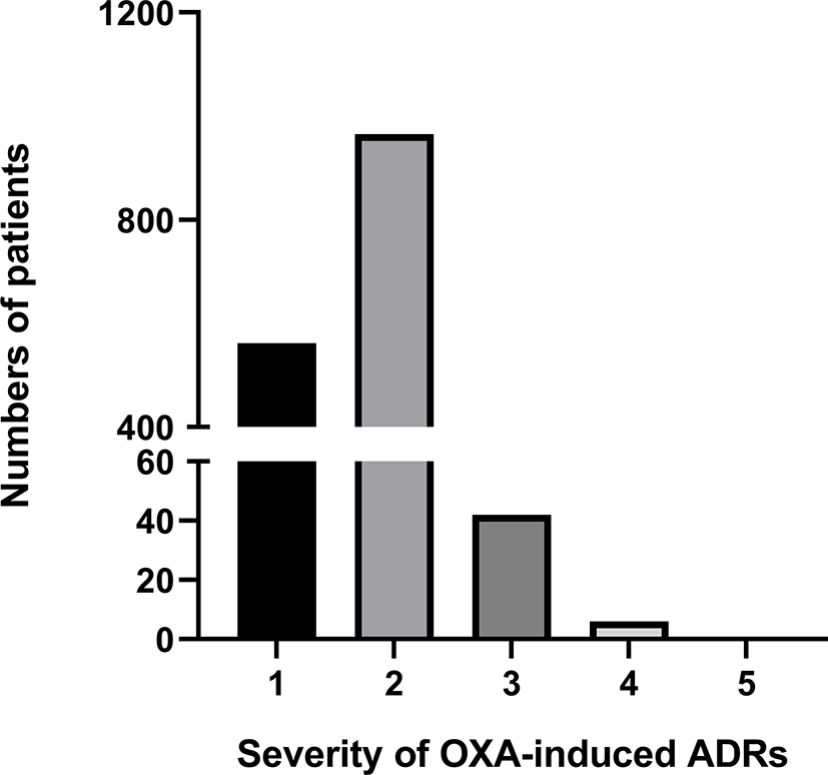
Severity classification of OXA induced ADRs.

Of the 3687 patients, ADRs were reported in 1575 patients, giving an incidence rate of 42.7%. Most ADRs were grade 1 and 2, with grade 1 ADR reported in 562 patients (15.2%) and grade 2 ADR reported in 965 patients (26.2%). Serious ADRs (grade≥3) were reported in 48 patients (1.3%), showed in **Figure 1**. The majority (88.0%) of patients who developed ADR were healed and improved, and about 11.0% were unknown.

The most commonly reported ADRs fell into the general categories of gastrointestinal disorders (25.7%), blood disorders (21.1%), and peripheral nervous system disorders (8.0%) (**Table 2**). Other ADRs, such as respiratory, cardiac and eye disorders, had a low incidence rate and low severity (**Table 2**). However, systemic disorders such as hypersensitivity reactions occurred in 118 of the patients (3.2%), with related serious ADRs accounting for 0.5% of all cases. In addition, hepatic function abnormalities were reported in 186 patients (5.1%), with serious ADRs of this nature accounting for 0.2% of all cases (**Table 2**).

**Table 2:** Incidence of ADRs and serious ADRs in patients receiving OXA (N = 3687).

System Organ Class	ADRs n (%)	Serious ADRs n (%)
Total	1575 (42.7)	48 (1.3)
Gastrointestinal disorders	949 (25.7)	10 (0.3)
Blood system disorders	779 (21.1)	18 (0.5)
Peripheral and Central Nervous system disorders	296 (8.0)	2 (0.0)
Systemic disorders and administration-site conditions	168 (4.5)	18 (0.5)
Hepatobiliary disorders	186 (5.1)	6 (0.2)
Respiratory disorders	11 (0.3)	2 (0.0)
Cardiac disorders	9 (0.2)	1 (0.0)
Eye disorders	4 (0.1)	0 (0.0)
Skin and subcutaneous tissue disorders	4 (0.1)	0 (0.0)
Musculoskeletal and connective tissue disorders	4 (0.1)	0 (0.0)
Renal and urinary disorders	3 (0.1)	0 (0.0)
Infections and infestations	1 (0.0)	1 (0.0)

### Most Frequently-Occurring ADRs

The OXA-linked ADRs with the highest frequency were nausea (20.3%), leukopenia (17.3%), neutropenia (12.1%), vomiting (9.8%), anemia (7.0%), thrombocytopenia (5.6%), and peripheral paresthesia or dysesthesia of hands and feet (5.7%). Meanwhile, the most frequent serious ADRs recorded were hypersensitivity reactions, leukopenia, neutropenia, thrombocytopenia, abnormal hepatic function, and vomiting (0.5%, 0.2%, 0.2%, 0.2%, 0.2%, 0.1%, respectively) (**Table 3**). Thus, gastrointestinal disorders, blood disorders, peripheral nervous system disorders, hypersensitivity reactions, and hepatic function abnormalities were regarded as the major ADRs caused by OXA.

**Table 3:** Incidence and severity of most frequently-occurring ADRs.

Major ADRs	ADRs n (%)	Serious ADRs n (%)
Gastrointestinal disorders		
Nausea	748 (20.3)	1 (0.0)
Vomiting	362 (9.8)	5 (0.1)
Diarrhea	71 (1.9)	2 (0.0)
Blood system disorders		
Leukopenia	637 (17.3)	8 (0.2)
Neutropenia	445 (12.1)	9 (0.2)
Anemia	258 (7.0)	2 (0.0)
Thrombocytopenia	207 (5.6)	6 (0.2)
Peripheral nervous system disorders		
Paresthesia or disesthesia (hands and feet)	211 (5.7)	0 (0.0)
Abnormal hepatic function	186 (5.1)	6 (0.2)
Hypersensitivity reactions	118 (3.2)	18 (0.5)

### Time to Onset, Management, and Outcome of Major ADRs

The median time from the start of OXA administration to the occurrence of gastrointestinal disorders, blood system disorders, peripheral nervous system disorders, and hepatic function abnormalities were 1 d, 5 d, 1 d, and 14 d, respectively. For hypersensitivity reactions, the median time from start to occurrence was much shorter, at only 20 min. In addition, the above 5 ADRs occurred in a median cycle of OXA chemotherapy of 3, 4, 4, 4 and 6, respectively. More than 90% of those who experienced gastrointestinal toxicity, myelosuppression, and peripheral neurotoxicity continued OXA therapy. Of those that experienced hypersensitivity reactions, approximately 85% suspended OXA administration and received the corresponding treatment (**Table 4**). Overall, most of these ADRs recovered and improved, and ADRs that had sequelae occurred in 11 patients who experienced hepatic dysfunction and 12 patients with myelosuppression.

**Table 4:** Time to onset, cycle, management and outcome of major ADRs.

Major ADRs	Median time to onset, days (range)	Median cycle of OXA chemotherapy (range)	Management of OXA- induced ADRs, % (n)		ADRs that recovered or improved, % (n)
			Continued	Suspended	
Gastrointestinal toxicity	1 (0–8)	3 (1–15)	94.2 (894)	3.2 (30)	94.8 (900)
Myelosuppression	5 (1–39)	4 (1–12)	92.8 (723)	3.6 (28)	82.5 (643)
Peripheral neurotoxicity	1 (0–7)	4 (1–15)	91.0 (242)	1.5 (4)	96.6 (257)
Abnormal hepatic function	14 (2–33)	4 (1–11)	95.9 (178)	3.2 (6)	77.5 (144)
Hypersensitivity reactions	20min (2-1440min)	6 (1–18)	14.4 (17)	85.6 (101)	100 (118)

### OXA-Related Hypersensitivity Reactions

The manifestation of OXA-related HSRs is shown in **Table 5**. The most common events were cutaneous symptoms such as flushing (48.3%), itching (48.3%), and rashes (22.9%). For most patients (84.7%), the symptoms were grade 1 or 2, while hypersensitivity symptoms with grade ≥ 3 were reported in 18 patients (15.2%). Of these 18 patients, six experienced anaphylactic shock, characterized by wheezing, dizziness, abdominal pain, or loss of consciousness with hypotension.

**Table 5:** Manifestations of OXA-induced hypersensitivity reactions.

	Subjects (total=118)		N (%)
Symptom	Cutaneous		
		Flushing	57 (48.3)
		Itching	57 (48.3)
		Rash/Urticaria	27 (22.9)
	Digestive		
		Nausea	35 (29.7)
		Vomiting	21 (17.8)
		Others	15 (12.7)
	Neurologic		
		Dizziness	7 (5.9)
		Numb	24 (20.3)
		Laryngeal abnormal	8 (6.8)
		Loss of consciousness	4 (3.4)
	Respiratory		
		Dyspnea	5 (4.2)
		Chest discomfort	45 (38.1)
		Wheezing	15 (12.7)
		Cough	2 (1.7)
	Generalized		
		Sweating	27 (22.9)
		Chills	3 (2.5)
		Fever	3 (2.5)
	Cardiovascular		
		Hypotension	13 (11.0)
		Tachycardia	21 (17.8)
	Vision		
		Blurred vision	2 (1.7)
		Conjunctival congestion	3 (2.5)
	Anaphylaxis		6 (5.1)
	Others		7 (5.9)
Grade of severity			
		1	24 (20.3)
		2	76 (64.4)
		3	12 (10.2
		4	6 (5.1)
The median time of hypersensitivity Reactions in different grades (min)			
		1	60 (10–1440)
		2	19 (2–180)
		3	10 (5–120)
		4	22 (2–60)
Management			
	Discontinuation of oxaliplatin administration		100 (84.7)
	Histamine-receptor 1 antagonist		61 (51.7)
	Dexamethasone		72 (61.0)
	Oxygen		34 (28.8)
	Subcutaneous adrenaline		10 (8.5)

**Figure 2:**
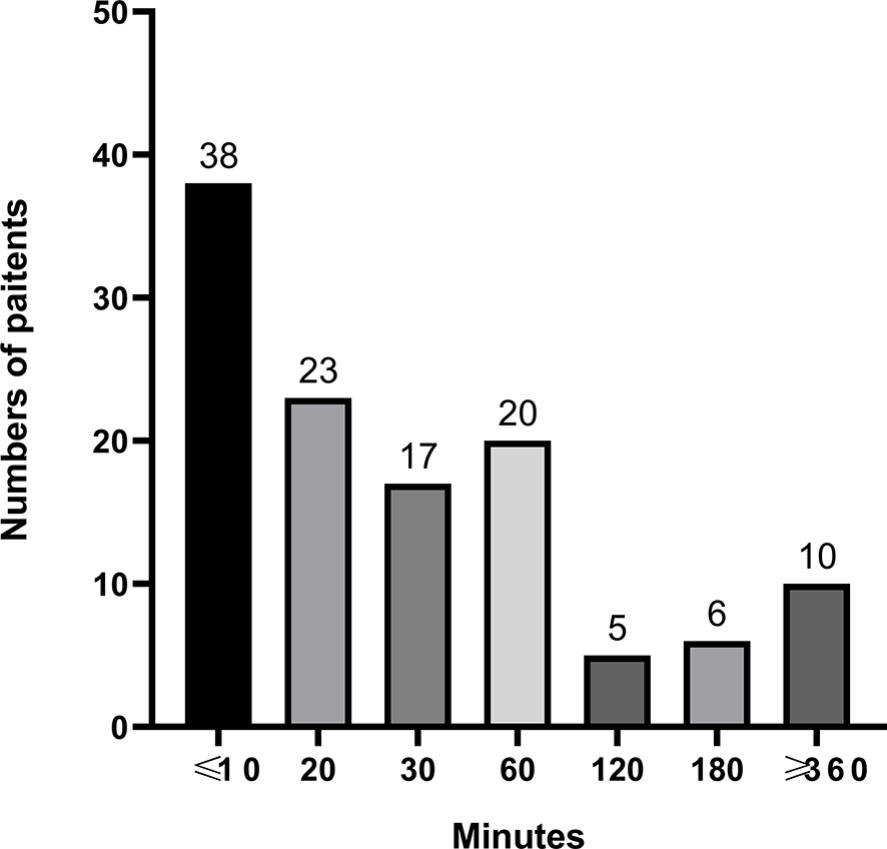
Occurrence time of HSR from the start of OXA infusion.

The time between OXA infusion to the appearance of HSRs is shown in **Figure 2**, with most reactions occurring within the first hour. The time to onset varied between mild and severe cases: the median time to onset of grade 1 HSRs was 60 min, while grade 2, 3, and 4 events occurred mainly within the first 20 min after OXA infusion (**Table 5**). Most patients who experienced hypersensitivity (84.7%) were managed *via* discontinuation of treatment with OXA and the administration of hypersensitivity treatments, including dexamethasone (61.0%), histamine-receptor 1 antagonist (51.7%), oxygen (28.8%), and epinephrine (8.5%). All patients recovered or improved after the corresponding symptomatic treatment (**Table 4**).

## Discussion

According to the 2018 Global Cancer Statistics, colorectal and gastric cancer incidence rates rank third and fourth among malignancies in China, and their mortality rate is placing fifth and second respectively (15). OXA, a third-generation platinum derivative, has become one of the mainstay chemotherapeutic drugs in gastrointestinal malignancies. The most common adverse reactions reported with this drug are gastrointestinal tract reactions, myelosuppression, peripheral neurotoxicity, and hypersensitivity reactions (14, 16). Hence, a multicenter observational study was carried out to investigate the safety profile of OXA in a real-world setting to provide a reference for the rational use of OXA.

In this study, 3687 patients who received OXA were enrolled. As far as we can confirm, at present, this is the largest real-world post-marketing safety evaluation of OXA in China. The majority (64.6%) of enrolled patients were male, within the age bracket of 50 to 69 years old. Epidemiology shows that the incidence rate of colorectal and gastric cancer in males is higher than in females, and most of these occur in middle-aged and older people (17). Thus, the characteristics of patients enrolled were consistent with previous epidemiological studies of colorectal and gastric cancer in China.

Moreover, this study showed that patients receiving OXA were mainly diagnosed with colorectal and gastric cancer (59.3% and 31.6%, respectively), and FOLFOX was the most commonly used chemotherapy regimen (61.7%), followed by XELOX (19.5%) and SOX (7.5%). According to NCCN guidelines of colorectal and gastric cancer (18, 19), FOLFOX and XELOX are the most popular chemotherapy regimens in colorectal cancer, which are widely used in neoadjuvant chemotherapy, adjuvant chemotherapy, and advanced palliative chemotherapy; XELOX and SOX are also frequently used chemotherapy regimens in gastric cancer. These suggest that the clinical application of OXA is in line with the recommendations described in these above guidelines.

Regarding the safety results, the overall incidence rate of ADRs was 42.7%, and that of serious ADRs was 1.3%. The most common reported ADRs of OXA in this study were gastrointestinal disorders (25.7%), blood disorders (21.1%), and peripheral nervous system disorders (8.0%), which were consistent with the results of other studies (14, 16, 20, 21).

Nausea, vomiting, and diarrhea were the most common gastrointestinal side effects of OXA, with a median time of onset of 1.6 d, and most patients recovered or improved quickly. Nausea and vomiting are usually mild to moderate and are readily controlled with the prophylactic administration of standard antiemetics such as dexamethasone or 5-HT3 receptor antagonists (22). Grade 1 and 2 diarrhea has been reported in OXA-treated patients with advanced colorectal cancer. The incidence of this ADR is usually higher with a protracted continuous infusion or with very high infusion doses (23). In practice, prophylaxis is not required, and the OXA dose should only be reduced in subsequent cycles if diarrhea becomes severe.

In general, hematological side effects caused by OXA include leukopenia, neutropenia, thrombocytopenia, and anemia (24). In our study, the prevalence of OXA-induced myelosuppression was 21.1%. Leukopenia (17.3%), neutropenia (12.1%), anemia (7.0%), and thrombocytopenia (5.6%) were also common hematological side effects of OXA treatment. Therefore, any patient treated with OXA should closely monitor their WBC count, platelet levels, hemoglobin levels, and absolute neutrophil count (ANC). Moreover, this study showed that thrombocytopenia was a common serious ADR, with an incidence of 0.2%, and the median time of onset from OXA treatment to this event was 4.7 d. Studies have demonstrated that thrombocytopenia was a prominent side effect of OXA-related myelosuppression (25, 26). Although thrombocytopenia of grades 3 and 4 was noted in only 3%-4% of patients exposed to OXA, its toxicity tends to increase with repeated exposures and may limit the benefits of OXA (25, 27). Recently, mechanisms of OXA-induced thrombocytopenia have emerged, including bone marrow suppression, immune-dependent mechanism, and splenic sequestration of platelets due to portal hypertension related to liver sinusoidal injury (11). However, in our study, it was difficult to determine the specific mechanism involved in OXA-induced thrombocytopenia; thus, this condition needs further study. Therefore, medical practitioners should be vigilant about thrombocytopenia caused by splenomegaly after long-term exposure to OXA and be aware of allergy-induced acute thrombocytopenia (28).

A major dose-limiting side-effect of OXA treatment is its peripheral neurotoxicity. Our study showed that OXA-induced peripheral neurotoxicity (OIPN) was reported in 266 patients (8.0%), mainly including paresthesias or dysesthesias of the hands and feet. Most reported OIPN cases were mild, and 96.6% of patients recovered or improved, with the median time to onset of 1 d. OXA induces two clinically distinct forms of peripheral neuropathy. The first is acute OIPN, which is transient, appearing only during or shortly after infusion of OXA, and can be triggered by cold stimulation. The other form, chronic OIPN, is associated with a cumulative dose of OXA and appears after administration of OXA at a total dose of 540-850 mg/m^2^ (12). Acute OIPN consists mainly of sensory symptoms in the form of paresthesias or dysesthesias in the distal or perioral regions and are related to the dosage and infusion rate of OXA. These symptoms are generally mild, short-lived, and completely reversible within a few hours or days (29). Thus, oncologists can prolong the duration of OXA infusion up to 6 h and request that their patients avoid cold liquids for several days after OXA therapy, which may prevent the development of acute OIPN.

While not as common as the previously discussed ADRs, hepatotoxicity also represents a serious ADR that warrants further investigation. There is evidence that the most common type of hepatotoxicity associated with OXA administration is hepatic sinusoidal injury. It is histologically characterized by sinusoidal dilatation, hepatocyte necrosis, and obliteration of hepatic venules due to sinusoidal endothelial cell damage (30–32). OXA-induced hepatic sinusoidal obstruction syndrome (HSOS) has been demonstrated in up to 77% of colorectal cancer patients with liver metastasis following OXA-based chemotherapy (33). Studies have reported that preoperative OXA was associated with HSOS and that this increased postoperative morbidity after partial hepatectomy with colorectal cancer liver metastasisn (34, 35). OXA-induced HSOS frequently presents with ascites, jaundice, right upper quadrant pain, splenomegaly with subsequent thrombocytopenia, and portal hypertension. Systemic elevation of liver enzymes is often not significant, especially in the early stage (30). We found that the incidence of abnormal hepatic function was only 5.1%; however, our study only measured hepatic biochemical parameters such as the levels of transaminases, aspartate aminotransferase, and bilirubin. Without the results of histopathological examinations, it is difficult to give a definitive diagnosis of HSOS, leading to an underestimation of the hepatotoxicity of OXA. Therefore, OXA-induced HSOS should be specially studied and evaluated in patients with metastatic colorectal cancer who received OXA-based neoadjuvant chemotherapy after hepatectomy.

OXA-related HSRs are another major problem associated with the extensive use of the drug, the occurrence of which may lead to therapy delay, discontinuation of treatment, and even death (36). The reported frequency of HSR in patients undergoing OXA-based chemotherapy ranges from less than 2% to 25% (37–42), while the prevalence of severe HSRs is 0.5-2% (38, 43). Our study reported OXA-related HSRs in 118 out of 3687 Chinese patients, giving an incidence rate of 3.2% and a severe hypersensitivity rate of 0.5%. The clinical manifestations of HSRs involve multiple systems, such as cutaneous, digestive, neurologic, and respiratory systems. This study found that the most common events were cutaneous symptoms with severities of grades 1 and 2, which corroborates the findings of our previous study (44). Events of grade 3 and above are less common, but six patients developed life-threatening cases of severe anaphylactic shock. These results align with other studies that reported OXA-induced HSRs being potentially severe and life-threatening (45). In this study, however, patients who developed HSRs were managed with the corresponding treatments, and eventually, all patients recovered or improved. This suggests that OXA-induced HSRs are controllable through close monitoring, comprehensive evaluation, and the provision of timely and effective treatment. Hence, medical staff should pay close attention to the signs of potential HSRs and inform patients to closely monitor any symptoms that may arise.

Previous studies have noted that OXA-induced hypersensitivity reactions usually occur within the first 30 min of infusion (46, 47). In our study, the median time to onset of HSRs was 20 min, but the time to onset of different grades of HSRs varied. The median time to onset of grade 1 HSRs was 60 min, while grade 2 to 4 events occurred mainly within the first 20 min of OXA infusion. Our previous retrospective analysis also demonstrated that HSRs caused by OXA might occur at any time within a cycle of therapy but were mainly observed at the first 20 min of OXA infusion (44). Thus, patients should be closely monitored for HSRs, but especially within the first 20 min after the start of an OXA infusion.

However, this study can still be further expanded. First, this was a non-interventional observational study that aimed to observe the safety profile of OXA with no control group, and there was a short observation period of only 6 months and lack of follow-up. Hence, the OXA efficacy in these patients was unknown, and some delayed ADRs such as chronic OIPN may not be immediately reported and were underestimated. Second, patients treated with OXA was the only inclusion criteria implemented, and a comparison of safety accounting for the effects of different diseases and treatment regimens was not performed. In addition, although the researchers have identified OXA-related ADRs, the effects of other drugs cannot be completely excluded because OXA is often used in combination with other chemotherapeutic drugs. Further investigation is also required to identify the risk factors that affect the incidence of OXA-induced ADRs to provide a reference for the rational application of OXA.

## Conclusions

In conclusion, this large post-marketing surveillance study conducted in more than 3000 Chinese patients preliminarily explored the incidence, characteristics, cycle, occurrence time and outcome of OXA induced ADRs. Overall, OXA induced adverse reactions were very prevalent. Our results showed that gastrointestinal toxicity, hematotoxicity, peripheral neurotoxicity, HSRs and abnormal liver function were the main common ADRs of OXA, in which the latter two had unique characteristics, need more attention, and warrant close monitoring during OXA infusion. Although further studies are still required, this study provides valuable reference for the rational use of OXA and has great guidance for the management of OXA-induced ADRs in routine clinical practice.

## Data Availability Statement

The original contributions presented in the study are included in the article/**Supplementary Material**. Further inquiries can be directed to the corresponding authors.

## Ethics Statement

The studies involving human participants were reviewed and approved by the Ethics Committee of Tongji Medical College, Huazhong University of Science and Technology (No.TJIRB20160504). The patients/participants provided their written informed consent to participate in this study.

## Author Contributions

ZY and RH, first authors, contributed to the data analysis and writing of manuscript. LZ and XY contributed to organization, management, and supervision of the project. XW and XS contributed to data collection, data curation, analysis, and validation. WL and ML contributed to contributed to the editing and submission of the article. CZ and DL, the corresponding authors, involved in conception of the project, performing data analysis, review of manuscript and provision of feedback and comments to first author. All authors contributed to the article and approved the submitted version.

## Funding

This work was supported by the National Natural Science Foundation of China (No: 7187040708), Hubei Center for Adverse drug reaction Monitoring (No: 20160422), Funding for research-oriented clinician plan of Tongji Medical College, Huazhong University of Science and Technology (No: 5001540076); Clinical toxicology foundation of Chinese Society of Toxicology (CST2020CT107).

## Conflict of Interest

The authors declare that the research was conducted in the absence of any commercial or financial relationships that could be construed as a potential conflict of interest.

## Publisher’s Note

All claims expressed in this article are solely those of the authors and do not necessarily represent those of their affiliated organizations, or those of the publisher, the editors and the reviewers. Any product that may be evaluated in this article, or claim that may be made by its manufacturer, is not guaranteed or endorsed by the publisher.
