# Supplementary material for: Safety Profile of Oxaliplatin in 3,687 Patients With Cancer in China: A Post-Marketing Surveillance Study
Source: Front Oncol. 2021 Oct 21;11:757196. doi: 10.3389/fonc.2021.757196 (PMC8567037; doi:10.3389/fonc.2021.757196)
Supplement: Supplementary file 1 [file Table_1.doc]

**Supplementary Table 1. Case report forms (CRF) of Oxaliplatin(OXA) safety re-evaluation study**

| **Basic information of patient** | **Name:** | **Ward:** | | **Gender:**□1=male □2=female | **Admission NO:** |
| --- | --- | --- | --- | --- | --- |
|  | **Patient ID (optional^2^):** | | | **Date of birth:** year month day | |
|  | **Height:** cm | **Weight:** kg | | **Nationality:**□1=Han nationality □2=other | |
|  | **Prior adverse drug reaction/events:**□1=No □2=Unknown □3=Yes  **Family adverse drug reaction/events:**□1=No □2=Unknown □3=Yes | | | | |
|  | **History of food allergy:**□1=No □2=Unknown □3=Yes, allergen: | | | | |
|  | **Other diseases:**□1=No □2=Allergic disease □2.1=Allergic rhinitis □2.2=Allergic asthma  □2.3=Allergic dermatitis □2.4= Allergic gastroenteritis □2.5=Other allergic diseases:  □3=Diabetes □4=Hypertension □5=Hyperlipidemia □6=Coronary heart disease □7=Other: | | | | |
| **The admission situation** | **Admission time:** year month day **KPS score:** scores | | | | |
|  | **Clinical diagnosis(Specific to staging):** | | | | |
| **Medication records** | **Drug information** | | **Manufacturer:** | | |
|  |  |  | **Specification:**  **Batch NO:** | | |
|  | **OXA chemotherapy cycle** | | □1=1 □2=2 □3=3□4=4 □5=5 □6=6 □7=Other: | | |
|  | **OXA administration rate**  **(Optional 1 item)** | | Starting and ending time of administration: month day hour minute to hour minute | | |
|  |  |  | Dripping speed Drops/min or ml/min(Used to record pumping time) | | |
|  | **Dosage and administration** | | OXA:Dosage mg；  Solvent □1=5%Glucose injection, Volume ml  □2=Other: Volume ml | | |
|  | **Chemotherapy Regimen** | |  | | |
|  | **Whether to use dexamethasone before OXA chemotherpy** | |  | | |
|  | **Other medications to prevent adverse reactions** | |  | | |
|  | **Concomitant medication** | |  | | |
| **OXA induced Adverse Reactions (ADRs)** | **The patient has the following adverse reactions:(After sub-items, the specific values and options are written according to the most serious situation)**  □**1=Blood system**  □1.1=Anemia (Hemoglobin g/L)  □1.2=Leukopenia ( ×10^9^/L)  □1.3=Neutropenia ( ×10^9^/L)  □1.4=Thrombocytopenia ( ×10^9^/L)  □1.5=Other  □**2=Gastrointestinal**  □2.1=Nausea (□ Does not affect eating □Eating less □Need nasal feeding or total parenteral nutrition)  □2.2=Vomiting, Attack every 24h (5 minutes intervals) □1-2 times□3-5 times □6 times and above  □2.3=Diarrhea, Daily increase over its own base □Less than 4 times □4-6 times □7 times and above  □2.4=Abdominal pain, Pain score scores □Affect sleep □ Need to use analgesics  □2.5=Constipation (□Occasional laxatives or enemas □Continued need for laxatives or enemas □Stubborn constipation needs manual dredging)  □2.6=Indigestion (□No treatment needed □Needs treatment)  □2.7=Flatulence(□No treatment needed □Needs treatment)  □2.8=Mucositis/Stomatitis  □2.9=Other  □**3=Peripheral nervous system** (□Does not affect function □Affect function, but not life □Affect life □Disabling or fatal)  □3.1=Paresthesia or dysesthesia of hands and feet  □3.2=Spasms and paresthesia around the mouth  □3.3=Throat paresthesia(difficulty swallowing or breathing but no wheezing or wheezing)  □3.4=Mandibular spasm □3.5=Tongue paraesthesia □3.6=Dysphonia □3.7=Eye pain □3.8=Chest pressure □3.9=Other  □**4=Local reactions at the injection site**  □4.1=Pain □4.2=Redness □4.3=Swelling □4.4=Thrombosis □4.5=Other  **□5=Allergic reaction**  □5.1=Rash (including urticaria) □5.2=Skin flushing □5.3=Pruritus □5.4=Hypotension □5.5=Bronchospasm □5.6=Facial flushing □5.7=Allergic diarrhea (occurs with OXA administration) □5.8=Shortness of breath □5.9=Sweating □5.10=Chest pain □5.11=Disorientation □5.12=Syncope □5.13=Increased eosinophils □5.14=Other allergic reactions:  **If an allergic reaction occurs, please select an allergic reaction classification**  □Grade I: Transient flushing or skin rash; drug fever <38℃, no need for treatment.  □Grade II: Skin rash or dyspnea requires intervention or infusion therapy; rapid symptomatic treatment (such as antihistamines, NSAIDs, anesthetics) can take effect.  □Grade Ⅲ: Significant bronchospasm with or without urticaria; need for intravenous treatment; angioedema/allergic edema; hypotension. The curative effect of symptomatic treatment is not rapid; initial symptoms relapse after improvement; sequelae (such as renal failure, lung infiltration) require the prolonged hospitalization.  □Grade Ⅳ: Acute allergic reactions are life-threatening, such as rapid rubella, respiratory depression, vascular collapse, shock, etc., requiring emergency treatment.  □Grade Ⅴ: Death.  □**6=Visual system**  □6.1=Visual abnormalities □6.2=Conjunctival abnormalities □6.3=Conjunctival mutation □6.4=Eyes weekness □6.5=Other: Visual field defect, blurred vision  □**7=Systemic abnormalities**  □7.1=Anorexia □7.2=Fatigue □7.3=Fever □7.4=Cold □7.5=Other:  □**8=Pain**  □8.1=Myalgia □8.2=Backache □8.3=Arthralgia □8.4=Bone pain □8.5=Other:  □**9=Central nervous system**  □9.1=Insomnia □9.2=Depression □9.3=Anxiety □9.4=Convulsion □9.5=Dizziness □9.6=Headache □Other:  □**10=Other**  □10.1=Alopecia  □10.2=Abnormal renal function  □10.3=Abnormal hepatic function  □10.4=Coagulation disorders, concrete manifestation  □10.5=Infection:(□Agranulocytosis with fever □Shock)  □10.6=Pulmonary Toxicity(□ Cough □ Dyspnea □ Interstitial lung disease □ Fibrosis)  □10.7=Reversible posterior leukoencephalopathy syndrome (Signs and possible symptoms: headache, mental changes, seizures, blurred vision to blindness, with or without high blood pressure. Can be diagnosed by brain imaging)  □10.8=Hemolytic uremic syndrome  □10.9=Other:  **Adverse reaction/event process description and treatment (required items):**      **The outcome of the patient's adverse reactions:**  □1=Healed □2=Improved □3=has sequelae, the sequelae are:  □4=Death, direct cause of death:  **The impact on primary illness:**  □1=Not obvious ¨2=Prolonged course of illness □3=Exacerbation of illness □4=Sequelae □5=Death | | | | |
| **ADR evaluation**  **(The clinical pharmacist should refer to the "Work Manual" to fill in)** | 1.ADR correlation  ADR relevance evaluation:   1. Whether there is a reasonable time relationship between medication and the occurrence of adverse reactions? □1=Yes □2=No □3=Uncertain 2. Whether the reaction is consistent with the known adverse reaction type of the drug? □1=Yes □2=No □3=Uncertain 3. Whether the reaction disappears or decreases after drug withdrawal or dose reduction? □1=Yes □2=No □3=Uncertain 4. Whether the same reaction occurs again when using suspicious drugs again □1=Yes □2=No □3=Uncertain 5. Whether the response can be explained by the effect of concomitant drugs, the progress of illness or other treatment measures □1=Yes □2=No □3=Uncertain   ADR relevance conclusion:  □1=definitely relevant □2=probably relevant □3=may be relevant □4=may not be relevant □5=to be evaluated □6=unable to evaluate  2. ADR severity classification: □1=Grade 1 □2=Grade 2 □3=Grade 3 □4=Grade 4 □5=Grade 5 | | | | |
| **Vital signs and laboratory tests**  **(Before OXA medication)** | 1.Vital signs month day hour minute:  BP: mmHg P: bpm R: times/min T: ℃  2.Blood routine(check date month day)  Hemoglobin(g/L) RBC(×10^12^/L) WBC(×10^9^/L) Platelets(×10^9^/L) Neutrophils(%) Eosinophils(%)  3.Coagulation function(check date month day)  Prothrombin time(s) Partial rothrombin time(s) Fibrinogen(g/L)  INR D-dimer (mg/L)  4.Renal function(check date month day)  Urea nitrogen(mmol/L) Creatinine(μmol/L) Uric acid(μmol/L)  5.Liver function(check date month day)  ALT(U/L) AST(U/L) ALP(U/L)  Total bilirubin(μmol/L) Direct bilirubin(μmol/L) Indirect bilirubin(μmol/L)  6.Other: | | | | |
| **Vital signs and laboratory tests** | 1.Vital signs month day hour minute:  BP: mmHg P: bpm R: times/min T: ℃  2.Blood routine(check date month day)  Hemoglobin(g/L) RBC(×10^12^/L) WBC(×10^9^/L)  Platelets(×10^9^/L) Neutrophils(%) Eosinophils(%)  3.Coagulation function(check date month day)  Prothrombin time(s) Partial rothrombin time(s) Fibrinogen(g/L)  INR D-dimer (mg/L)  4.Renal function(check date month day)  Urea nitrogen(mmol/L) Creatinine(μmol/L) Uric acid(μmol/L)  5.Liver function(check date month day)  ALT(U/L) AST(U/L) ALP(U/L)  Total bilirubin(μmol/L) Direct bilirubin(μmol/L) Indirect bilirubin(μmol/L)  6.Other: | | | | |
| **Remark** | If there are other important relevant information about this patient, please fill in here: | | | | |

Filler’s signature: Date of filing: / / /
